# Efficacy of cranial electrotherapy stimulation in patients with burning mouth syndrome: a randomized, controlled, double-blind pilot study

**DOI:** 10.3389/fneur.2024.1343093

**Published:** 2024-02-14

**Authors:** Annalena Palmer, Till Hamann, Jan Liese, Britta Müller, Peter Kropp, Tim P. Jürgens, Florian Rimmele

**Affiliations:** ^1^Department of Neurology, Headache Centre North-East, University Medical Centre Rostock, Rostock, Germany; ^2^Department of Oral, Maxillofacial and Facial Plastic Surgery, University Medical Centre Rostock, Rostock, Germany; ^3^Institute of Medical Psychology and Medical Sociology, University Medical Centre Rostock, Rostock, Germany; ^4^KMG Hospital Güstrow, Güstrow, Germany

**Keywords:** facial pain, headache, burning mouth syndrome (BMS), cranial electrotherapy stimulation (CES), transcutaneous electrical nerve stimulation (TENS), Glossodynia, chronic pain, neuropathic pain

## Abstract

**Background:**

The Burning mouth syndrome (BMS) is a chronic pain syndrome characterized by a burning sensation in the oral mucous membranes. The etiology and pathophysiology of BMS is largely unexplained. To date, there is no evidence-based treatment strategy for BMS. Cranial electrical stimulation (CES) represents a non-invasive treatment option with a low side effect profile that is approved for the treatment of pain, depression, anxiety disorder and insomnia. It has shown efficacy in studies for chronic pain such as fibromyalgia and neuropathic pain after spinal cord injury. This study aimed to investigate the therapeutic effectiveness of CES in combination with local transcutaneous electrical nerve stimulation (TENS) as an adjunct therapy in patients with BMS compared to sham stimulation.

**Methods:**

This randomized, double-blind, sham-controlled pilot study enrolled 22 patients, aged 18 years and over, with the diagnosis of BMS meeting the ICHD-3 criteria from August 2020 to June 2021. The study duration was 4 weeks (28 days) per participant. After randomization, the active group participants (*n* = 11) received a 100 μA CES treatment for 60 min a day whereas the devices in the Sham group did not emit electricity. Simple linear regression was used to determine whether the interventions promoted significant differences in pain intensity.

**Results:**

The linear regression showed that the period of stimulation significantly predicted decrease in the intensity of pain in the active group [*β* = −0.036; *t*(26) = −7.219; *p* < 0.001] as in the sham group [*β* = −0.026; *t*(26) = −2.56; *p* < 0.017]. With the applied cutoff of 30% pain reduction within the stimulation period, both the active and sham groups had 36% responders (*n* = 4) (Fisher’s exact test, *p* = 1.00). In both groups (active stimulation and sham group), a significant decrease in the intensity of pain, somatic symptoms and an improvement in sleep quality over the study period was observed. Subjects reported no adverse events during the study.

**Conclusion:**

Although CES is an easily applicable and safe therapeutic option for chronic facial pain, active stimulation was not superior to sham stimulation. Among other reasons, this could be due to the short double-blinded treatment period, duration of the daily stimulation session or the small sample size.

## Background

Burning mouth syndrome (BMS) is a poorly understood chronic pain disorder characterized by an intraoral burning sensation in the absence of any identifiable organic cause according to the International Headache Society classification (ICHD-3) (1).

The prevalence of BMS in the population ranges from 0.7 to 15%, depending on the diagnostic criteria used, with postmenopausal women being more frequently affected (2). The average age of BMS patients is 61 years. It is assumed that there is a male-to-female ratio of 1:5–1:7, and the prevalence seems to increase with advancing age in both genders (3, 4).

According to the current ICHD-3 classification the burning pain is felt superficially in the oral mucosa, recurring daily for more than 2 h per day over more than 3 months. In the physical examination, the oral mucosa has a normal appearance and clinical findings including sensory testing are normal (1).

The painful sensation predominantly affects the tongue (67.9%), with the anterior two-thirds of the tongue being most commonly affected. However, it may also extend to other regions of the oral mucosa, including the floor of the mouth and the lips (5). The pain experienced typically ranges from moderate to severe intensity, quantified within a range of 3.1–5.11 on the Numeric Rating Scale (NRS) (5). The most frequently described accompanying symptoms are a dry mouth (xerostomia), described in 46–70% of cases, followed by taste disturbances (5). Psychosocial and psychological comorbidities manifest in 85% of cases, with anxiety disorders and depression being particularly common (2). BMS Patients often report a bad sleeping quality (6). The consumption of foods with high acidity, spiciness, or temperature exacerbates the discomfort. Chewing gum and mouth rinses use to alleviate the pain (5).

The etiology and pathophysiology of BMS remains mostly unknown. Pathophysiological concepts include peripheral and central mechanisms. Neurophysiological concepts include damage to the chorda tympani, damage to the lingual nerve (a branch of the trigeminal nerve that supplies the oral mucosa), peripheral small-fiber polyneuropathy, altered cortical networks involving the “pain matrix” and descending inhibitory pathways as well as impaired dopaminergic inhibition (1, 6). A biopsy study by Lauria et al. on 12 BMS patients demonstrated a significant reduction in epithelial and subpapillary nerve fibers in the lingual mucosa, which strengthens the hypothesis of small-fiber polyneuropathy (7). There also appears to be a strong interaction with psychological factors, especially depression and anxiety disorders (8, 9).

On the other hand, “secondary” BMS is attributed to identifiable local or systemic factors. Local factors include odontogenic diseases, mechanical and chemical irritants, viral, bacterial, or fungal infections, as well as hypersensitivity reactions. Systemic factors may be induced by drugs, anemia, vitamin B12 or folic acid deficiency, Sjögren’s syndrome, and diabetes mellitus (2). In this study, we adopted the ICHD-3 classification, in which BMS is diagnosed only after ruling out all potential local and systemic causes. BMS-like symptoms that can be attributed to the aforementioned factors should be labeled as symptomatic BMS and be treated with a targeted therapy for the causing factor (1).

A therapeutic strategy for BMS based on evidence from studies is missing. The treatment approaches can be differentiated into symptomatic and topical therapies. Topical treatments include capsaicin and benzodiazepines, systemic treatments pregabalin, gabapentin or tricyclic antidepressants like amitriptyline. Given the large psychiatric comorbidity, cognitive behavioral approaches complement pharmacologic treatments. Multimodal, multidisciplinary therapy with pharmaceutical and psychosocial approaches seems to be most effective. However, the evidence regarding the success of these treatment options is limited (2, 5). Cranial electrical stimulation (CES) is a non-invasive procedure involving transcutaneous application of pulsed, low amplitude (<1 mA) electrical voltage via electrodes to the earlobes. CES received FDA (Food and Drug Administration) approval in 1979 for the treatment of depression, anxiety disorder and insomnia (10). The exact mechanism of action of CES is unknown. Studies have shown that CES can affect the blood levels of various neurotransmitters, such as beta-endorphin or serotonin, and may act via the limbic system, the reticular ascending system (RAS) and the hypothalamus (11, 12). Changes in the EEG after CES use have been observed, including an increase in alpha and a decrease in beta and delta activity. These alterations indicate a possibly improved relaxation. CES also appears to influence the brain’s default mode network (DMN) (13), which is significantly activated by serotonin and whose connectivity is altered in depression, sleep disorders, anxiety disorders and pain (13, 14). Many studies have investigated the benefits and effectiveness of CES. Also, in randomized, double-blind, controlled clinical trials, a positive effect for CES on pain was shown in patients with fibromyalgia (15) and in neuropathic pain after spinal cord injury (12). There is also supporting evidence for the amelioration of psychological comorbidities, which are highly prevalent among individuals with BMS, through CES. In a randomized, double-blind, controlled clinical trial, Barclay and Barclay showed significant improvement after CES in patients with anxiety disorder and comorbid depression (11).

In Transcutaneous electrical nerve stimulation (TENS) electrical currents are delivered to the skin through surface electrodes for pain relief.

The precise mechanism of TENS is not fully understood. Its analgesic effect is believed to be complex, involving peripheral, spinal, and supraspinal mechanisms. Animal studies demonstrated that the analgesic effect was partly mediated by peripheral mechanisms like a decreased peripheral response to serotonin and changes in antinociceptive α2A-adrenergic receptors (16). A spinal effect for electrical stimulation was initially demonstrated by Melzack and Wall’s Gate Control Theory which suggests that afferent fibers can inhibit nociceptive activity in the dorsal horn of the spinal cord, resulting in reduced pain perception (17). Animal studies also indicate changes in neurotransmitter levels like GABA and Glycine associated with TENS (18, 19). Regarding central mechanisms, there is a suggestion that TENS stimulates descending inhibitory nerve pathways and increases the release of endorphins (20). Positive effects of TENS on pain have been demonstrated in patients with fibromyalgia (21) and neuropathic pain after spinal cord injury (22). Additionally, TENS has shown efficacy as a therapeutic option in trigeminal neuralgia, migraine, and cluster headache (23–27).

In this study we aimed to investigate the therapeutic effectiveness of CES in combination with local TENS as an adjunct therapy in patients with BMS compared to sham stimulation. We assessed the impact on pain intensity, sleep quality, and psychological comorbidities, such as somatic symptoms disorder, depression and anxiety disorders.

## Methods

### Participants

Subject recruitment was carried out at the University Medical Centre Rostock through the Department of Oral, Maxillofacial, and Facial Plastic Surgery and the Headache Centre North-East. Inclusion criteria included a physician-diagnosed BMS according to the ICHD-3 criteria of the IHS, a minimum age of 18, stable pain and antidepressant medication for at least 1 month (or the absence of such medication), and the commitment of subjects not to alter their medication during the study. Exclusion criteria included active implants (e.g., pacemakers, defibrillators), pregnancy or lactation, and limited contractual capacity.

### Intervention

The study utilized “Alpha-Stim M” devices from “Electromedical Products International, Inc.” Fifty percent of the devices provided active stimulation with a current of 100 μA and 0.5 Hz, while the remainder served as sham stimulators without any current emission. Patients applied daily 60-min CES to the auricular lobules, along with a 3-min TENS of the tongue, over a 28-day period. To monitor in-home stimulation, we employed a tracking system, and patients were required to document the execution and effects of the stimulation in their pain diary daily.

### Design

**Figure 1:**
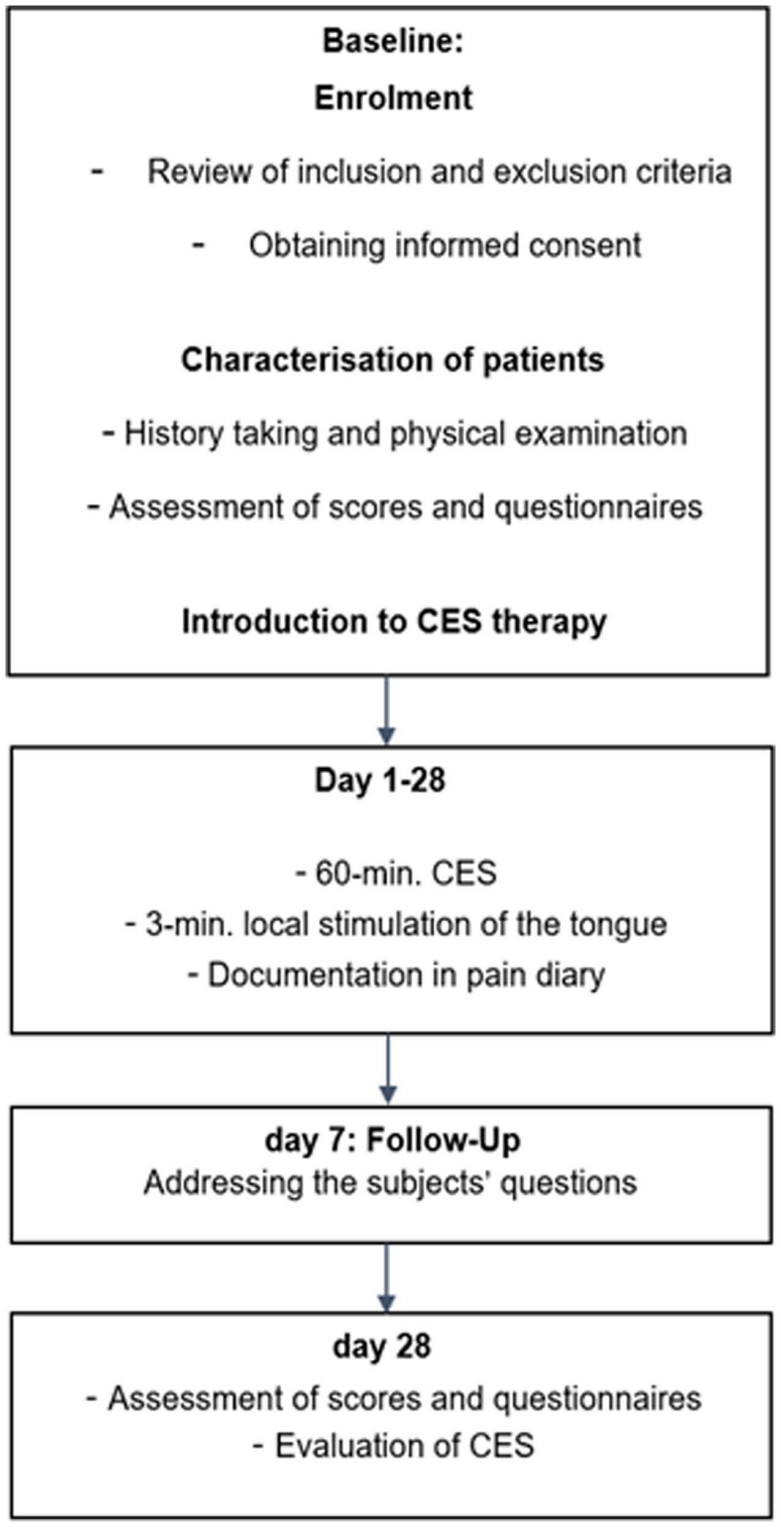
Study timeline.

At the initial appointment, subjects were extensively briefed on the study procedure, potential side effects, and risks of CES in a medical consultation. After assessing the inclusion and exclusion criteria and obtaining written consent, subjects received an introduction to CES stimulation and were guided through a self-administered trial stimulation. Furthermore, at the baseline (day 0) and the end of the study (day 28), patients completed the following questionnaires: The Pittsburgh Sleep Quality Index (PSQI) was utilized for sleep assessment, the Oral Health Impact Profile (OHIP-14) served as an oral health evaluation, and for the assessment of somatic symptoms, the Somatic Symptom Module of the Patient Health Questionnaire (PHQ) was employed. To evaluate anxiety and depression, the following questionnaires were utilized: the Patient Health Questionnaire for Depression (PHQ-D), Hamilton Rating Scale for Depression (HAMD), Hamilton Anxiety Rating Scale (HAMA), and Hospital Anxiety and Depression Scale (HADS). The study duration was 4 weeks (28 days) per subject. A paper diary was used daily to characterize pain. The reported daily maximum pain score on the NRS before stimulation served for subsequent evaluations of the stimulation’s impact on pain, which is the primary outcome of this study. Additionally, interference, pain-amplifying and alleviating factors, accompanying symptoms, and medication usage were documented. Furthermore, the diary recorded details about the stimulation, including its effects and side effects. After 1 week (Day 7), a follow-up appointment was conducted to inquire about the subjects’ use of CES and address any questions they might have had. Due to the COVID-19 pandemic, this appointment was conducted via telephone. After the 4-week study period, a final evaluation meeting took place to assess the stimulation, review the pain diary, and readminister the questionnaires. The study timeline is visualized in Figure 1.

### Outcomes

The primary outcome of the study was to explore therapeutic effects of CES on pain in patients with BMS, in comparison to sham stimulation. As a secondary outcome, the study investigated its effects on sleep quality and mental health.

### Statistical analyses

The SPSS version 27 statistical package for Microsoft Windows was used to analyze the data (28). Qualitative variables were represented by their absolute (n) and relative (%) frequencies, mean and standard deviation. Homogeneity of the study groups on day 0 (baseline) in their variables was tested with t-tests. Simple linear regression was used to evaluate the association between duration of stimulation (measured in days) and pain intensity (measured in the NRS score). Changes of scores a two-way ANOVA with repeated measures was used to examine the interaction of time (baseline and day 28) and group (real stimulation and sham stimulation). To compare the rate of responders Fisher’s exact test was used. A probability of less than *p* ≤ 0.05 was regarded as significant.

### Blinding

Blinding was carried out by the company and was only disclosed to the clinical staff upon completion of the study for data analysis. The devices shared identical appearances and controls, rendering them indistinguishable externally.

### Ethical aspects

The research was conducted in accordance with the declaration of Helsinki and approved by the Ethics committee of the University Medical Center Rostock (A2020-0138). All study participants were provided with written information about the study procedure prior to inclusion in the study and gave their informed consent before participating in the study.

Pseudonymization was implemented.

## Results

**Figure 2:**
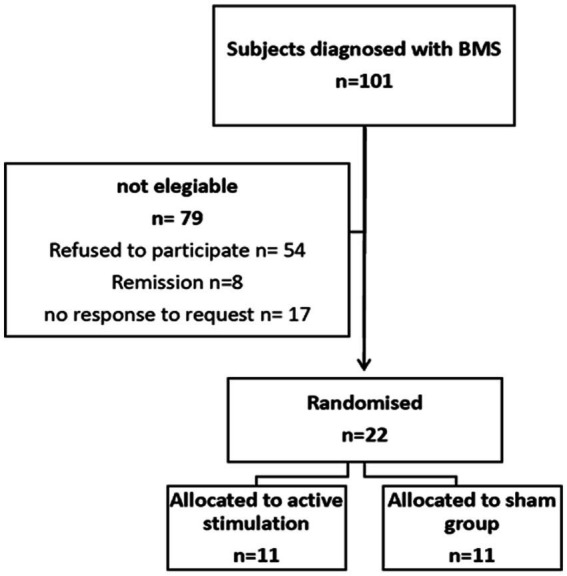
Enrolment of patients.

The process of patient recruitment is shown in Figure 2. At the onset of recruitment, there were 101 potential study participants with BMS. Out of these, 22 patients met the inclusion criteria and none of them discontinued the study. The average age of the entire group was 64 years, with 77% of the patients being female. Demographic and clinical characteristics of patients are summarized in Table 1.

**Table 1:** Demographic and clinical characteristics of patients.

Characteristics		Stimulation group (*n* = 11)	Sham group (*n* = 11)	Total (*n* = 22)
Sex female, (%)		85.7	88.9	87.5
Age in y, mean (SD)		63.09 ± 13.69	63.09 ± 8.71	63.09 ± 2.3
Duration of illness in years, mean (SD)		5.95 ± 7.29	6.20 ± 5.63	6.07 ± 1.35
Medication, (%)	Pregabalin	9.1	0	4.5
	NSAR for facial pain	0	18.2	9.1
	Antidepressant medication	18.2	0	9.1
Oral and maxillofacial disorders, (%)	None	81.8	72.7	68.2
	HO dental surgery	9.1	27.3	18.2
	Craniomandibular dysfunction	9.1	0	4.5
Neurological disorder (%)	None	63.6	72.7	68.2
	Episodic migraine	27.3	9.1	18.2
	Tinnitus	9.1	9.1	9.1
	HO stroke	0	9.1	4.5
Diagnosed psychiatric disorders, (%)	None	81.8	90.9	86.4
	Depression	18.2	0	9.1
	Somatic symptom disorder	0	9.1	4.5
Metabolic disorders, (%)	Morbus Fabry	9.1	0	4.5
	Hypothyroidism	0	9.1	4.5
Rheumatic diseases, (%)	Rheumatoid arthritis	0	9.1	4.5
	CREST-syndrome	9.1	0	4.5
Cardiovascular diseases, (%)	Arterial hypertension	36.4	63.6	50
Musculoskeletal disorders, (%)	Spinal stenosis	9.1	18.2	13.6
	Herniated disc	9.1	9.1	9.1
	Leg length discrepancy	9.1	0	4.5
	Osteoarthritis	9.1	0	4.5
Gastrointestinal disorders, (%)	HO gastritis	0	27.3	13.6
	Gastroesophageal reflux	18.2	0	9.1
Gynecological Comorbidities, (%)	HO carcinoma	0	18.2	9.1
	HO hysterectomy	18.2	0	9.1
Chemotherapy, (%)	HO chemotherapy	0	9.1	4.5

HO, “history of”.

### Pain

**Figure 3:**
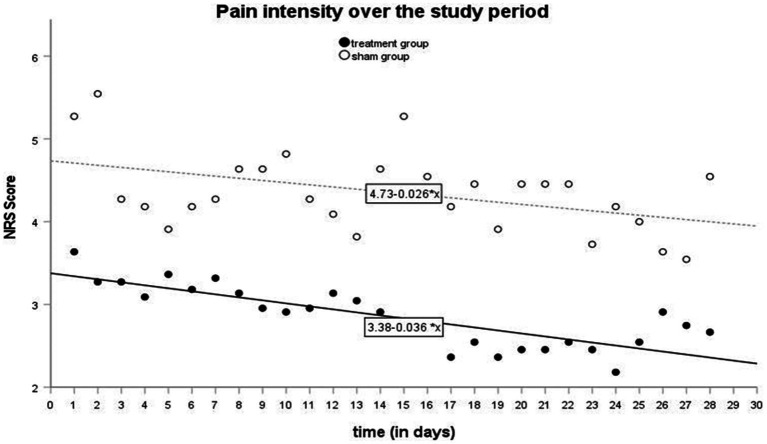
Pain intensity over the study period.

Pain was mostly described as a burning sensation in the tongue, with a moderate intensity (M ± SD = 4.50 ± 2.3). Nineteen out of the 22 patients reported daily pain localization in their headache diaries, with the tongue being the most frequently affected location (63%, *n* = 12). During patient history assessments, all 22 patients described their pain as “burning.” There were no significant differences between the active and sham groups. The most frequently mentioned accompanying symptoms were xerostomia, taste disturbances, and sensory distortions. Most frequent pain triggers reported by patients included spicy, acidic, hot, sweet and cold food and stress. The most frequently mentioned pain-relieving factors were chewing gum, mouth rinses food and fluid intake (Supplementary Tables S1, S2). Simple linear regression showed that the period of stimulation significantly predicted decrease in the intensity of pain in the active group [*β* = − 0.036; *t*(26) = −7.219; *p* < 0.00] as in the sham group [*β* = − 0.026; *t*(26) = −2.56; *p* < 0.017]. The results are visualized in Figure 3 and summarized in Table 2. An identification of responders and non-responders was conducted. Using the applied cut-off of 30% pain reduction at the end of the stimulation period, both the active and sham groups had 36% responders (*n* = 4) (Fisher’s Exact Test, *p* = 1.00).

**Table 2:** Regression analyses summary (Association between duration of stimulation and pain intensity).

	Stimulation group			Sham group		
	Unstandardized		Standardized	Unstandardized		Standardized
	B	SE	Beta	B	SE	
Constant	3.377***	0.084		4.734***	0.170	
Day	−0.036***	0.005	−0.817***	−0.026**	0.010	−0.449**
*R* ^2^	0.667			0.201		
Adj. *R*^2^	0.654			0.171**		
F (df 1; 26)	52.110***			6.552		

**p < 0.05; ***p < 0.001.

### Psychiatric comorbidities

Patients had a high prevalence of psychiatric comorbidities. The prevalence of somatic symptoms was high. 45% (*n* = 10) of the subjects had mild somatic symptoms, 18% (*n* = 4) moderate somatic symptoms and 23% (*n* = 5) severe somatic symptoms. In the overall study population, a statistically significant decrease in the somatic symptom disorder scores over the study period [*F*(1, 20) = 4.91; *p* = 0.039; η^2^ = 0.20] was observed. However, no significant difference was observed between the active and sham stimulation [*F*(1, 20) = 0.24; *p* = 0.628; *η*^2^ = 0.01] Applying the official cut-offs, 41% (*n* = 9) had a mild anxiety severity, 14% (*n* = 3) a moderate anxiety severity, and one patient had severe anxiety symptoms. No statistically significant change in the anxiety scores was observed over the study period [*F*(1, 20) = 0.86; *p* = 0.364; *η*^2^ = 0.04]. 41% (*n* = 9) of the subjects were mild depressed and 18% (*n* = 4) were moderate depressed. No statistically significant change of depression was observed over the study period [*F*(1, 20) = 0.80; *p* = 0.381; *η*^2^ = 004]. There was no statistically significant difference between the groups at the baseline for the assessed scores.

### Sleeping disorders

Compared to the general population the sleeping quality was poorer. According to the general cut-off. Only 23% (*N* = 5) had a good sleeping quality, 50% (*N* = 11) had a bad sleeping quality and 27% (*N* = 6) have a clinically relevant sleep disorder. The subjects in the active group had a better sleeping quality at baseline [*t*(20) = −2.75; *p* = 0.012]. The sleeping quality statistically significant improved in both groups [*F*(1, 20) = 13.21, *p* < 0.05; *η*^2^ = 0.40]. However, there was no difference between the active and the sham group [*F*(1, 20) = 3.06; *p* = 0.095; *η*^2^ = 0.13].

### Oral health

The examined BMS patients had poor oral health-related quality of life. There was no statistically significant difference between the groups. The values were at the 80th percentile or higher for all three categories of dental status. There was no statistically significant improvement in oral health in both groups.

### Patient’s evaluation

The results in the evaluation regarding improvements in pain, sleep quality, psychological well-being, and overall improvement ranged between categories (3) “no change” and (4) “mild improvement” in both groups. There were no statistically significant differences between the groups in any of these variables. The patients were “neither nor” and “moderately satisfied” with CES. Consequently, only 28% of both the sham and active groups would recommend CES as a treatment option.

### Tolerability and safety

None of the patients reported severe adverse events during TENS or CES. Stimulation was never discontinued due to side effects. The most common side effect was localized tingling.

## Discussion

CES represents a non-invasive treatment option with a low likelihood of severe adverse events. Over the study period, both groups experienced a significant reduction in pain. However, superiority of active stimulation over sham stimulation could not be demonstrated. Other studies have shown a significant reduction in pain intensity through CES in chronic pain syndromes, such as fibromyalgia or pain in Parkinson’s disease (15, 29, 30). TENS as well has demonstrated efficacy in individuals (21), neuropathic pain after spinal cord injury (22) and various types of headaches in clinical studies (23–27). As TENS and CES share similarities in their application, mechanisms, and effects, there is a potential for a synergistic analgesic effect. Additionally, there could be an additional local effect through the TENS stimulation directly on the tongue, where the main symptoms of BMS are located. However, in our study, the patients’ evaluation at the end of the study period indicates that the modest improvements were not significant enough to create a subjective sense of improvement for the majority of the patients. Many BMS patients had a long history of unsuccessful treatments, leading to a high burden of disease and therefore high hope for an improvement. When this improvement does not occur in the expected intensity, it can reinforce the already felt frustration. It is known that BMS patients tend to catastrophize (31). Catastrophizing has been defined as an exaggerated negative orientation toward pain stimuli and pain experience (32). It affects the modulation of pain stimulus, the way patients cope with their pain, and the response to the treatment (33). These aspects could have been a reason that the patients did not perceiving little changes and evaluated the stimulation rather negative. The subjects for the study were recruited via the Headache Centre and the Department of Oral and Maxillofacial Surgery at Rostock University Medical Centre, so that a selected clientele was probably included in the study. The patients were often severely affected and had remained refractory to previous treatment attempts.

Another factor that influenced the results is the placebo effect, which is often particularly strong in patients with pain and can pose a methodological problem in the control group, especially in stimulation studies. In other BMS studies, a significant placebo effect has been reported, ranging from 15 to 75% (34). Barcley et al. conducted a 5-week randomized, double-blind, placebo-controlled study to evaluate the efficacy of CES for various anxiety disorders and comorbid depression. Patients in the active group received 60 min of stimulation over the course of 5 weeks, and their symptoms were assessed using the HAMA and HAMD. They found a significant decrease of anxiety and comorbid depression scores in the active group. However, a significant increase of 28% in anxiety scores was also observed in the sham group. The authors attributed this result to the placebo effect (11).

Also in chronic migraine, another chronic pain condition with a similar spectrum of especially psychiatric comorbidities, a substantial placebo effect is often observed. The reported placebo effect in the acute treatment of migraine attacks is up to 47% and in migraine prophylaxis usually between 20 and 40% (35).

The choice of sham stimulation can pose a methodological problem, as shown in the study by Straube et al. who investigated the efficacy of transcutaneous stimulation of the auricular branch of the vagus nerve in chronic migraine patients. Active stimulation with a 1 Hz frequency was used in the control group, which surprisingly proved to be more effective than the stimulation at 25 Hz in the active group. The authors initially expected the 25 Hz stimulation to be more effective, and the 1 Hz frequency was primarily planned for blinding purposes only. It remains unclear whether this result was due to a placebo effect or if the frequency was already high enough to induce a therapeutic effect (36). Although we chose inactive devices for the control group to avoid this effect in our study, including a non-intervention group would have provided additional information for both studies. As it helps to evaluate whether the stimulation process itself induced the reduction in pain intensity or a placebo effect. As a secondary endpoint, our study investigated the impact of CES on psychiatric comorbidities and sleep. At the baseline a greater number of patients in the active group were utilizing antidepressants and pregabalin. Nevertheless, considering the limited potential for neuromodulation devices to interfere with concurrent treatments or associated comorbidities, this is expected to have a relatively modest effect (10, 37, 38). Despite the presence of high psychosomatic comorbidity in our study population, there was no significant decrease in depression and anxiety scores between the beginning and end of the study either. This differs from numerous other studies that have described CES as a good treatment option for psychiatric conditions (10, 39, 40). However, contradictory and heterogeneous results are also described in the literature. In a Cochrane Review, O'Connell et al. concluded that the evidence was insufficient to support the use of CES for depression due to the low quality of the studies (41). The FDA also reached a similar conclusion in December 2019 stating that effectiveness of CES for the treatment of depression was “unclear.” They noted significant limitations in the quality of available clinical studies and a lack of high evidence results regarding the use of CES in patients with depression or sleep disorders (42). In summary, the evidence regarding the effectiveness of CES for psychiatric conditions is heterogeneous and does not allow a definitive judgment. Kirsch and Gilula conclude in a meta-analysis on the use of CES for insomnia that CES is an excellent treatment option for patients with insomnia. The review examined 20 studies using CES for the treatment of patients with primary insomnia, as well as with insomnia occurring as a comorbidity with psychiatric and pain-related disorders (39). We observed an improvement in sleep quality in some patients. However, in comparison to other studies, the observed effect was small, and no advantage of active stimulation over sham stimulation could be observed.

### Limitations

As the study was designed as a pilot study, we considered the sample size of 22 as sufficient given the difficult recruiting during the COVID19 pandemic. Nevertheless, this small sample size limits the interpretation of the results. This is a common problem in studies with CES, so the literature on the effects of CES is dominated by small studies with unclear risk of bias (41, 42). The study was carried out by the Headache Centre Rostock in collaboration with the Department of Oral, Maxillofacial, and Plastic Surgery in Rostock. The University Medical Centre Rostock serves as care provider for the region, and these centers serve as the primary points of contact and consultation for BMS patients. However, as the BMS is often a non-diagnosed syndrome, it was a rather small number of subjects which was eligible for the study (*n* = 101). To aim for a larger patient population, it would be necessary to either screen for BMS patients regionally in smaller centers such as dental practices or cooperate with other centers.

The study’s design was created similarly to other related studies regarding stimulation parameter to enhance comparability. The stimulation intensity of 0.5 Hz and 100 μA has been employed in numerous other studies (11, 29, 43). At this current intensity, 1 h of application is recommended, as was the case in the present study (43). The sham devices had the identical appearance, and it is not possible to distinguish from the active devices. Stimulation at the intensities used is below the threshold of perception, so that the participants should not be able to differentiate between active and sham conditions (41, 44). Patients could have been asked at the end of the study which study group they believed they were in. This could have provided insight into whether the sham stimulation provided adequate blinding. A possible reason for the small observed effect could be that the chosen study period of 4 weeks was too short. It appears that the impact of stimulation on pain intensity accumulates over time. While there was no indication of a short-term effect immediately after stimulation, both groups experienced a significant reduction in pain over the study period. Furthermore, considering that the stimulation resulted in a greater reduction of pain in the active group compared to the passive group, an extended study period could reveal a significant difference. This is supported by the observation that individuals with depression and sleep disorders exhibit a slower response to CES compared to the overall study population of patients with psychological disorders. Assessment points at three and 6 weeks of study duration have been recommended (10). A study by Holubec states that CES has a positive cumulative effect on refractory patients in patients with a variety of pain-related disorders (45). Our study did not have a follow-up session after the stimulation. Scheduling another appointment a month after the end of the study would provide additional information to evaluate long-term effects.

## Conclusion

This study aimed to investigate the therapeutic effectiveness of CES as an adjunct therapy in patients with BMS compared to sham stimulation. According to our study results, CES is a low-risk and easily applicable therapeutic option that led to an improvement in symptoms and reduced the burden of the disease in some BMS patients. Over the study period, both groups experienced a significant reduction in pain intensity, somatic symptoms and an improvement in sleep quality. Superiority of active stimulation over sham stimulation could not be demonstrated. To prove the high effectiveness of CES, further studies with strong evidence are necessary. Studies exploring the ethology of BMS are needed to develop therapeutic approaches, as well as clinical trials aimed at devising improved treatment strategies for BMS.

## Data availability statement

The original contributions presented in the study are included in the article/Supplementary material, further inquiries can be directed to the corresponding author.

## Ethics statement

The studies involving humans were approved by the Ethics Committee of the University Medical Centre Rostock. The studies were conducted in accordance with the local legislation and institutional requirements. The participants provided their written informed consent to participate in this study.

## Author contributions

AP: Data curation, Formal analysis, Investigation, Writing – original draft. TH: Formal analysis, Writing – review & editing. JL: Formal analysis, Writing – review & editing. BM: Formal analysis, Writing – review & editing. PK: Formal analysis, Writing – review & editing. TJ: Conceptualization, Formal analysis, Writing – review & editing. FR: Conceptualization, Formal analysis, Writing – original draft, Writing – review & editing.
