# Supplementary material for: Efficacy of cranial electrotherapy stimulation in patients with burning mouth syndrome: a randomized, controlled, double-blind pilot study
Source: Front Neurol. 2024 Feb 14;15:1343093. doi: 10.3389/fneur.2024.1343093 (PMC10900232; doi:10.3389/fneur.2024.1343093)
Supplement: Supplementary file 1 [file Data_Sheet_1.docx]

**Supplementary Tables**

Table 1: Homogeneity of the study groups at baseline (T-test, day 0)

| T-Test  Homogeneity of the study groups on day 0 |  | |  |
| --- | --- | --- | --- |
|  | M± SD | |  |
| Scores | Stim. group  (n=11) | Sham group  (n=11) | t |
| Age | 63.09 ± 13.69 | 63.09 ± 8.71 | 0.000 |
| Duration of illness in years | 5.95 ± 7.29 | 6.20 ± 5.63 | - 0.088 |
| NRS | 3.91 ± 2.26 | 5.09 ± 2.30 | - 1.216 |
| SFMPQ2-Total | 9.82 ± 9.06 | 9.36 ± 5.84 | 0.140 |
| SFMPQ-Sens | 52.00 ± 21.11 | 62.27 ± 21.26 | - 0.153 |
| SFMPQ-Aff | 5.18 ± 3.43 | 8.55 ± 4.70 | 0.835 |
| PDI Sum | 9.09 ± 3.96 | 11.27 ± 6.69 | - 2.992* |
| EQ5D3L TTO Score | 0.68 ± .26 | 0.68 ± .07 | - 2.253* |
| EQ5D VAS Score | 52.00 ± 21.11 | 62.27 ± 21.26 | 1.110 |
| PHQ-D | 9.91 ± 4.53 | 10.55 ± 7.10 | - 1.918 |
| PHQ-15 | 8.36 ± 5.99 | 10.82 ± 6.88 | - 0.930 |
| HADS anxiety scores | 6.27 ± 3.00 | 10.00 ± 3.35 | 0.000 |
| HADS depression score | 5.18 ± 3.89 | 6.73 ± 4.29 | - 0.885 |
| HAMA Score | 9.91 ± 4.53 | 10.55 ± 7.10 | - 0.251 |
| HAMD | 8.36 ± 5.99 | 10.82 ± 6.88 | - 0.892 |
| TAS-26 Index | 40.00 ± 12.00 | 40.27 ± 6.84 | - 0.065 |
| PSQI | 6.27 ± 3.00 | 10.00 ± 3.35 | - 2.749* |
| OHIP | 45.00 ± 38.89 | 47.36 ± 28.96 | - 0.162 |
| *p < 0.05 |  |  |  |

Table 2: Response to therapy by treatment and group (ANOVA with repeated measures)

| ANOVA with repeated measures  Response to therapy by treatment and measurement | | | | |  | | | |  | |  | |
| --- | --- | --- | --- | --- | --- | --- | --- | --- | --- | --- | --- | --- |
|  | T0 | | | | T2 | | | |  | |  | |
| Scores | Stim. group  (N=11) | | Sham group  (N=11) | | Stim. Group  (N=11) | | Sham group  (N=11) | | F  time | | F  time*  Group | |
| NRS | 3.91 ± 2.26 | | 5.09 ± 2.30 | | 3.00 ± 1.61 | | 4.36 ± 1.36 | | 4.53* | | 0.056 | |
| SFMPQ2-Total | 9.82 ± 9.06 | | 9.36 ± 5.84 | | 9.18 ± 7.64 | | 9.00 ± 6.03 | | 0.22 | | 0.02 | |
| SFMPQ-Sens | 8.00 ± 6.69 | | 8.36 ± 4.18 | | 6.91 ± 6.58 | | 7.55 ± 4.41 | | 1.29 | | 0.03 | |
| SFMPQ-Aff. | 1.82 ± 2.52 | | 1.00 ± 2.05 | | 1.64 ± 1.86 | | 1.64 ± 1.86 | | 0.11 | | 0.62 | |
| PDI Sum. | 11.36 ± 10.33 | | 30.13 ± 18.12 | | 9.73 ± 9.07 | | 25.09 ± 16.51 | | 3.43 | | 0.90 | |
| EQ5D3L-TTO | .68 ± 0.26 | | .68 ±0.07 | | .71 ± 0.26 | | .86 ± 0.05 | | 0.14 | | 0.16 | |
| EQ5D-VAS | 52.00 ± 21.11 | | 62.27 ± 21.26 | | 44.00 ± 17.76 | | 62.27 ± 13.30 | | 1.16 | | 1.16 | |
| PHQ-D | 9.91 ± 4.53 | | 10.55 ± 7.10 | | 10.09 ± 4.95 | | 8.27 ± 5.82 | | 3.05 | | 0.212 | |
| PHQ-15 | 8.36 ± 5.99 | | 10.82 ± 6.88 | | 7.91 ± 7.06 | | 8.82 ± 5.90 | | 4.91* | | 0.24 | |
| HADS anxiety | 6.55 ± 3.80 | | 6.55 ± 3.24 | | 5.64 ± 3.50 | | 6.91 ± 3.70 | | 0.37 | | 1.99 | |
| HADS depression | 5.18 ± 3.89 | | 6.73 ± 4.29 | | 4.91 ± 4.35 | | 6.91 ± 4.50 | | 0.01 | | 0.30 | |
| HAMA | 9.91 ± 4.53 | | 10.55 ± 7.10 | | 10.09 ± 4.95 | | 8.27 ± 5.82 | | 0.86 | | 1.19 | |
| HAMD | 8.36 ± 5.99 | | 10.82 ± 6.88 | | 7.91 ± 7.06 | | 8.82 ± 5.90 | | 0.17 | | 0.80 | |
| TAS-26 Index | 2.22 ± 0.67 | | 2.26 ± 0.32 | | 1.73 ± 0.52 | | 1.82 ± 0.55 | | 7.61* | | 0.02 | |
| PSQI | 6.27 ± 3.00 | | 10.00 ± 3.35 | | 5.64 ± 2.69 | | 8.18 ± 3.25 | | 13.21* | | 3.06 | |
| OHIP | 45.00 ± 38.89 | | 47.36 ± 28.96 | | 37.64 ± 37.06 | | 44.45 ± 23.14 | | 3.80 | | 0.71 | |
| *p < 0.05 |  |  | |  | |  | |  | |  | |  |
